# Effects of Second Dose of SARS-CoV-2 Vaccination on Household Transmission, England

**DOI:** 10.3201/eid2901.220996

**Published:** 2023-01

**Authors:** Asad Zaidi, Ross Harris, Jennifer Hall, Sarah Woodhall, Nick Andrews, Kevin Dunbar, Jamie Lopez-Bernal, Gavin Dabrera

**Affiliations:** COVID-19 Vaccines and Epidemiology Division—UK Health Security Agency, London, UK (A. Zaidi, J. Hall, S. Woodhall, G. Dabrera);; Statistics, Modelling, and Economics Department—UK Health Security Agency (R. Harris, N. Andrews);; Institute for Women’s Health, University College, London (J. Hall);; Immunisation and Countermeasures Division—UK Health Security Agency (K. Dunbar, J. Lopez-Bernal)

**Keywords:** COVID-19, SARS-CoV-2, severe acute respiratory syndrome coronavirus 2, viruses, respiratory infections, zoonoses, transmission, household, vaccination, United Kingdom, *Suggested citation for this article*: Zaidi A, Harris R, Hall J, Woodhall S, Andrews N, Dunbar K, et al. Effects of second dose of SARS-CoV-2 vaccination on household transmission, England. Emerg Infect Dis. 2023 Jan [*date cited*]. https://doi.org/10.3201/eid2901.220996

## Abstract

A single SARS-CoV-2 vaccine dose reduces onward transmission from case-patients. We assessed the potential effects of receiving 2 doses on household transmission for case-patients in England and their household contacts. We used stratified Cox regression models to calculate hazard ratios (HRs) for contacts becoming secondary case-patients, comparing contacts of 2-dose vaccinated and unvaccinated index case-patients. We controlled for age, sex, and vaccination status of case-patients and contacts, as well as region, household composition, and relative socioeconomic condition based on household location. During the Alpha-dominant period, HRs were 0.19 (0.13–0.28) for contacts of 2-dose BNT162b2-vaccinated case-patients and 0.54 (0.41–0.69) for contacts of 2-dose Ch4dOx1-vaccinated case-patients; during the Delta-dominant period, HRs were higher, 0.74 (0.72–0.76) for BNT162b2 and 1.06 (1.04–1.08) for Ch4dOx1. Reduction of onward transmission was lower for index case-patients who tested positive ≥2 months after the second dose of either vaccine.

Observational studies conducted in several countries including Qatar (*1*) and the United Kingdom (*2*) have demonstrated that SARS-CoV-2 vaccination provides protection against symptomatic infection, hospitalization, and death. The role of SARS-CoV-2 vaccination in preventing transmission from persons with confirmed infection to others has also been demonstrated previously using household contacts as a high-risk exposure group (*3*,*4*). Previous work conducted in England in early 2021 using the Household Transmission Evaluation Dataset (HOSTED) identified reduced odds of infection among unvaccinated household contacts of vaccinated cases compared with those for unvaccinated household contacts of unvaccinated cases (*3*). We studied the period in which the interval between vaccine doses for adults was as long as 12 weeks when the national program could focus on rapidly establishing maximum single-dose coverage for the population (*5*). Consequently, most adults had received 1 dose of vaccine and the available information was insufficient to investigate whether additional doses of vaccine might yield further benefits in reducing transmission. In that period, the Alpha variant emerged and rapidly became the dominant SARS-CoV-2 variant in England; household transmission was greater than that of previously circulating SARS-CoV-2 (*6*). Since then, the epidemiologic situation has changed significantly with the emergence of new variants, the expansion of the national vaccine rollout program and subsequent increase in population coverage of second and booster doses, and changes to public health measures. Those factors all underline the need for further investigation and analysis. The objective of our study was to expand our initial assessment of the effect of SARS-CoV-2 vaccine on transmission to household contacts to include 2 doses of vaccine and incorporate the period dominated by the Delta variant.

## Methods

### Dataset Creation

The creation of the routine HOSTED dataset has been described in detail (*3*,*7*). In brief, test-confirmed cases of COVID-19 in England are reported to national laboratory surveillance systems (*8*). During the period studied, the widely used assays were the reverse transcription PCR (RT-PCR) and the lateral flow device (LFD) test; national requirements to confirm a positive LFD test with a RT-PCR test and the presence of a negative confirmatory RT-PCR removed the initial positive tests from the data. Reported cases are linked to a Unique Property Reference Number (UPRN) via healthcare records associated with their National Health Service (NHS) number. The UPRN is an identifier allocated to each dwelling in England and can be used to assess property class (residential, commercial, etc.) and property type (flat, semi-detached, terraced, etc.). The cases’ UPRNs are then used to extract data on persons who share the same residence and may or may not themselves be cases.

Because the aim of our analysis was to estimate effects on transmission within the most common residential household groupings, we included only residential dwellings that had 2–10 residents in the dataset. We excluded single-resident households, households with >10 residents, and known institutional settings such as care homes and prisons.

We linked the HOSTED dataset with data from the National Immunisation Management System (*9*) in England to obtain information for all persons within the cohort on the dates and types of COVID-19 vaccination doses administered. The final dataset included specimen dates of cases; individual-level sociodemographic data for case-patients and those listed as residing alongside case-patients, including age and sex; household-level sociodemographic data such as Index of Multiple Deprivation (IMD), household size, and property type; individual-level outcome information on hospitalizations and deaths; and individual-level vaccination information on the date and type (ChAdOx1 [AstraZeneca, https://www.astrazeneca.com], BNT162b2 [Pfizer-BioNTech, https://www.pfizer.com], or mRNA-1273 [Moderna, https://www.modernatx.com]) of first and second doses for all vaccinated persons in the dataset.

### Definitions

Using the chronology of specimen dates within each household, we defined index cases as the earliest case of test-confirmed COVID-19 for a household. We defined household contacts as all persons registered to live at the same UPRN as each index case of COVID-19. We defined secondary cases as a known household contact of an index case who had a positive SARS-CoV-2 test with a specimen date 2–14 days after the specimen date of the index case.

We included contacts of index cases with a specimen date of February 1, 2021–September 13, 2021 in the analysis. We excluded households in which transmission patterns were likely different from household transmission in the community: households in which the index case was tested in the UK’s Pillar 1 system, which historically consists predominantly of hospital-based testing; households in which the index case was <16 years of age and ineligible for vaccination during the period studied; households with vulnerable residents who were vaccinated before the general rollout in the United Kingdom (January 4, 2021); and households with multiple index cases or co-primary cases.

We restricted our analysis to index cases and contacts with a clearly-defined vaccination status: unvaccinated persons who had not received any vaccine before the index case date and fully vaccinated persons who had received 2 doses of vaccine, the second dose >14 days before the index case date. Thus, we excluded all persons with 1 dose or other partial protection from the vaccine-related analysis, although we counted them in the definition of household size and composition variables. The period of study preceded the rollout of booster doses for the population by age criteria, which began in September 2021 for adults in risk groups and those >50 years of age and opened to all adults in November 2021.

### Statistical Analysis

We analyzed data in a time-to-event framework with household contacts at risk of being a secondary case 2–14 days after the specimen date of the index case. The main variable of interest was the vaccination status of the index case; we calculated adjusted hazard ratios for contacts of index cases who had received 2 vaccine doses versus contacts of unvaccinated index cases.

We fitted stratified Cox regression models with age of index case, age of contact, household type/size, region, and vaccination status of contact ​as strata variables. We adjusted for week of index case specimen date, deprivation (IMD quintile), sex of index case, and sex of contact. We fitted models separately to each calendar month of data, February–September 2021, and in 2 broad periods, Alpha-dominant (February–May 2021) and Delta-dominant (June–September 2021). 

We conducted 2 sensitivity analyses. The first included unvaccinated contacts only, as in previous analyses (*3*), to enable us to compare our findings with earlier estimates. The second excluded any persons (index cases or contacts) still unvaccinated as of September 13, 2021, when all the adult population would have been eligible and had the opportunity to be vaccinated, to exclude potential biases in case ascertainment for this group. We also obtained estimates from an unstratified cohort model, and included these strata variables as adjustment variables in the model.

In addition, we extended the base model to consider effect modification by dose interval, time since vaccination, and age. First, we divided the interval between first and second doses in the index case into 3 categories: <6 weeks, 6–10 weeks, and >10 weeks. We defined time since vaccination by 30-day categories (<30 days, 30–59, 60–89, 90–119, 120–149, and >150 days from second dose of index case vaccination to index case date) and compared vaccine transmission effects by both calendar time and time since vaccination. Finally, we considered the effect of age by estimating a separate vaccination transmission effect for each age group. We assessed extensions to the model via likelihood ratio tests.

## Results

The initial dataset comprised 1,779,448 index cases from 1,535,288 unique households, and 4,110,051 contacts. For those households in which transmission patterns were likely different from household transmission in the community, and to maintain comparability to the earlier analysis, we applied exclusions in 2 steps, first at the household level, and then at the individual level. For the first step we applied overlapping criteria, excluding households with index cases in children (n = 1,265,196 persons), with residents tested in pillar 1 (n = 99,627), with co-primary index cases (n = 785,669), and with residents who were vaccinated ahead of the national rollout (before January 2021; n = 194,160), leaving 1,035,271 index cases or households and 2,818,017 contacts. For the second step, we excluded persons with indeterminate vaccine status, after which there were 606,720 index cases and 1,440,269 contacts in the analysis dataset. Characteristics of contacts excluded through specific criteria compared with those excluded for indeterminate vaccination status of household members were similar in age, IMD, and geography (Appendix Table 1); the main difference appeared to be an increased proportion of secondary cases in households with multiple index cases.

The median age of index cases was 35 (IQR 25–50) years, and of contacts, 32 (IQR 13–52) years. We restricted index cases to those >16 years of age and eligible for vaccination, whereas we included all contacts. The proportion of female participants was 50.5% in index cases and 49.4% in contacts. We included 122,423 (8.5%) secondary cases among contacts (Appendix Table 2). During February–May 2021, proportions of secondary cases were highest in household contacts of unvaccinated index cases. However, proportions increased in contacts of index cases vaccinated with ChAdOx1 and BNT162b2 vaccines over time. From June onwards, these were highest among contacts of index cases who had received ChAdOx1 vaccine. Proportions of secondary cases among contacts of index cases receiving mRNA-1273 vaccine were consistently low, although data were sparse.

We estimated a strong protective effect for ChAdOx1 vaccine (HR 0.54, 95% CI 0.41–0.69) (Table) and a very strong effect for BNT162b2 vaccine (HR 0.19, 95% CI 0.13–0.28) in the February–May period, which persisted under different inclusion criteria and models. However, we estimated that in June–September the ChAdOx1 vaccine had minimal effect except in model D (Table), which included only unvaccinated contacts, and the protective effect of BNT162b2 vaccine was attenuated (HR 0.74, 95% CI 0.72–0.76). Estimates for mRNA-1273 vaccine, for which there were data only in June–September, were strongly protective.

**Table:** Adjusted hazard ratios for household contacts of persons with COVID becoming a secondary case by inclusion criteria for different models

Period	Vaccine	Analysis model HR (95% CI)				
		A, never vaccinated	B, not vaccinated before index case	C, vaccinated after index case	D, unvaccinated contacts only	E, cohort
Feb-May	ChAdOx1	0.54 (0.41–0.69)	0.50 (0.38–0.64)	0.50 (0.38–0.65)	0.48 (0.37–0.64)	0.53 (0.43–0.66)
	BNT162b2	0.19 (0.13–0.28)	0.18 (0.12–0.26)	0.18 (0.12–0.26)	0.14 (0.09–0.23)	0.19 (0.13–0.26)
Jun-Sep	ChAdOx1	1.06 (1.04–1.08)	1.06 (1.04–1.08)	0.94 (0.88–1.00)	0.84 (0.76–0.92)	1.08 (1.06–1.10)
	BNT162b2	0.74 (0.72–0.76)	0.74 (0.73–0.76)	0.66 (0.62–0.71)	0.56 (0.51–0.62)	0.76 (0.74–0.78)
	mRNA-1273	0.36 (0.29–0.45)	0.36 (0.29–0.45)	0.31 (0.25–0.39)	0.30 (0.22–0.40)	0.38 0.30–0.47)

*For models A–D, we used a stratified Cox model with adjustment, and different inclusion criteria for contacts and of the baseline group of contacts of unvaccinated index cases. Model E has the same inclusion criteria as model A, but uses a cohort model with all covariates entered as adjustment variables, rather than stratification. HR, hazard ratio.

We estimated the effect of index case vaccination over time and according to time since index case vaccination (Figure). For BNT162b2 vaccine, we noted strong protective effects up to 2 months after the index case was vaccinated, but hazard ratios were attenuated toward the null from 2–3 and 3–4 months after vaccination, particularly in August and September. The pattern of decreasing effect with time since index case vaccination was less clear for ChAdOx1 vaccine; we estimated little protective effect even soon after vaccination from June onwards. Estimates crossed the null to indicate an apparent increased risk of being a secondary case in August–September for contacts of index cases who had received ChAdOx1 vaccine, compared with contacts of unvaccinated index cases. 

**Figure:**
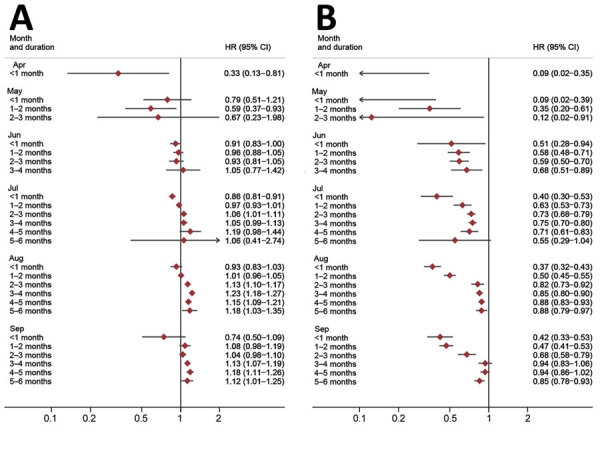
Adjusted hazard ratios for household contacts of COVID patients becoming secondary cases by calendar time and time since vaccination of the index case. A) Data for contacts of index cases who had ChAdOx1 vaccine (AstraZeneca, https://www.astrazeneca.com). B) Data for contacts of index cases who had BNT162b2 vaccine (Pfizer-BioNTech, https://www.pfizer.com). HR, hazard ratio.

## Discussion

This analysis provides evidence that persons who have received >2 doses of vaccine >14 days before testing positive for SARS-CoV-2 infection had a reduced likelihood for onward transmission to household contacts in February–May 2021, the period in which Alpha variant was dominant. Of note, this reduction was smaller for BNT162b2 vaccine and all but disappeared for ChAdOx1 vaccine during the subsequent June–September 2021 period, when the Delta variant dominated England. ChAdOx1 vaccine showed reduction in onward transmission only in household contacts who were completely unvaccinated. This finding is consistent with observations of reduced vaccine effectiveness against symptomatic disease for confirmed cases of Delta infection compared with Alpha (*2*,*10*–*12*).

mRNA-1273 vaccine played a smaller role in the initial vaccination rollout of primary courses, so we could only analyze the effects of the vaccine during the latter half of the study period. However, we noted a large and sustained reduction in household transmission in addition to the direct protection of the vaccines in preventing index cases initially and therefore potential for transmission. We also demonstrated a differential reduction in the likelihood of household transmission that is based on the type of vaccine. During the period of Delta dominance, mRNA-1273 vaccine had the greatest association with reduced likelihood of household transmission, followed by BNT162b2 vaccine and then ChAdOx1 vaccine. This finding was consistent with observations of higher vaccine effectiveness with mRNA-1273 and BNT162b2 vaccines compared with ChAdOx1 vaccine for the prevention of symptomatic infection in confirmed Delta variant infections (*2*). This triangulation with observations from other analyses of protection from disease (*2*) provides further evidence of a differential effect by vaccine for Delta variant cases.

Our work supplements the growing body of evidence related to waning of vaccine-driven protection. Previous analyses have shown reduced protection of persons from symptomatic infection (*10*,*11*). Our findings support another dimension to the waning effectiveness of vaccines: an attenuation in the reduction of onward transmission from index cases vaccinated with Ch4dOx1 and BNT162b2 vaccines to their household contacts (*13*). We observed this effect for vaccinated index cases who tested positive for COVID-19 >2 months after receiving their second dose. Hazard ratios for becoming a secondary case increased among household contacts of those case-patients, compared with the contacts of index cases who had a shorter interval between their vaccination and confirmed infection. The protective effect against onward transmission conferred by ChAdOx1 vaccine was initially smaller than that for BNT162b2 vaccine and diminished to no effect as time since second dose increased. The protective effect conferred by BNT162b2 vaccine, despite also reducing over time, did not fully disappear. Waning among index cases receiving mRNA-1273 vaccine could not be analyzed in this way because this vaccine was rolled out later in England.

This waning effect was more consistently observed from July 2021 onward, before which the effect was present but less apparent. Early July 2021 demarcates the transition between periods of Alpha and Delta variant dominance in England, suggesting a potential intrinsic difference between the variants and related protection from the vaccine (*13*).

Because vaccination programs have evolved internationally to provide boosters to vulnerable groups, we may observe a temporary reversal of the waning protection against onward transmission from cases to others. However, this effect requires further evaluation; factors such as time interval between doses, prevalent variants, and vaccine types could all influence any potentially observed effects. Such evaluation would be informative for future assessments of the overall effects of booster programs and therefore influence decision making.

The HOSTED analysis benefits from its very large number of cases and their contacts obtained from data for all reported test-confirmed cases in England; the dataset provides substantial sample sizes that enable large-scale monitoring of secondary infections without specialized data collection. In addition, we conducted several sensitivity analyses, which produced consistent and convergent results. However, the tradeoff is that passive surveillance systems are reliant on testing behaviors instead of targeted case-finding. In passive surveillance, chains of onward household transmission may be missed where contacts did not test, and the secondary attack rate would be underestimated as a result. These missed chains of transmission would be disproportionately present in households where access to testing is lower because of circumstance or choice. This result might explain our observation that contacts of index cases vaccinated with ChAdOx1 with a vaccination-infection interval of >3 months since their second dose had an apparent increase in risk compared with contacts of unvaccinated index cases in August–September 2021. Although vaccination might not reduce onward transmission, increased infectivity caused by vaccination does not appear biologically plausible. This increased risk for infection in the contacts of vaccinated index cases likely indicates differential case ascertainment or risk behaviors between vaccinated and unvaccinated groups, demonstrating a limitation of passive surveillance data.

Without specialized data collection, we were limited by the lack of information on symptoms and case severity, both of which may affect the likelihood of onward transmission (*14*). As such, our analyses treated all test-confirmed infections uniformly; the granularity that symptom and severity data provide could have highlighted subpopulations for which the vaccine-induced reduction of household transmission was more or less noticeable than for the overall population.

The logic underpinning how cases in a household are classified as index or secondary cases is rooted in the intervals between specimen dates, an approach already established in published analyses. However, the use of symptom onset dates may be more optimal, with symptom onset dates potentially being less affected by behavior and health service access than specimen collection dates.

In conclusion, receipt of 2 doses of mRNA vaccines among index cases reduced transmission to unvaccinated household contacts during a period in which the Delta variant dominated, compared with the Alpha-dominant periods, during which a single dose of mRNA or ChAdOx1 vaccine provided equivalent reductions in transmission. We also observed waning of this protective effect over time since the last dose of vaccine administered. These findings highlight the potential variation in protection from vaccines in relation to transmission effects that are caused by emerging variants and waning protection, and the importance of monitoring the effects of these factors for future SARS-CoV-2 vaccine strategies.

Appendix Additional information about the effects of a second dose of SARS-CoV-2 vaccination of index cases on household transmission, England.
