# Supplementary material for: Effects of Second Dose of SARS-CoV-2 Vaccination on Household Transmission, England
Source: Emerg Infect Dis. 2023 Jan;29(1):127–32. doi: 10.3201/eid2901.220996 (PMC9796187; doi:10.3201/eid2901.220996)
Supplement: Appendix — Additional information about the effects of a second dose of SARS-CoV-2 vaccination of index cases on household transmission, England. [file 22-0996-Techapp-s1.pdf]

Article DOI: https://doi.org/10.3201/eid2901.220996

Effects of Second Dose of SARS-CoV-2 Vaccination on Household Transmission, England

Appendix

Appendix Table 1. Characteristics of household contacts included and excluded from analysis of the effects of SARS-CoV-2 vaccination on household transmission

Characteristic	Included data	Excluded: characteristic-based	Excluded: indeterminate vaccine status
Percentage of secondary cases	8.5	11.1	7.0
Age, median y (IQR)	33 (14–52)	33 (13–45)	32 (18–50)
% F	49.4	50.2	49.5
Mean IMD decile	5.1	5.3	5.4
Region row percentages			
North	37.5	35.8	26.7
Midlands	37.6	35.7	26.7
London	39.6	32.0	28.4
South	35.8	32.9	31.3
Calendar time row percentages			
Feb–Mar	45.7	34.5	19.8
Apr–May	29.6	40.9	29.5
Jun–Jul	34.2	31.5	34.3
Aug–Sep	39.1	37.3	23.7

*Values are % except as indicated. IMD, Index of Multiple Deprivation; IQR, interquartile range.

Appendix Table 2. Characteristics of household contacts of index cases in study of effects of a second dose of SARS-CoV-2 vaccination on household transmission, England*

Characteristic	Unvaccinated		Ch4dOx1 nCOV-19		BNT162b2		mRNA-127		Total	
	Contacts	Secondary cases	Contacts	Secondary cases	Contacts	Secondary cases	Contacts	Secondary cases	Contacts	Secondary cases
Age of index case, y										
16–24	327,113 (37.8)	21,743 (6.6)	48,140 (11.3)	2,394 (5.0)	22,481 (15.3)	687 (3.1)	175 (7.3)	1 (0.6)	397,909 (27.6)	24,825 (6.2)
25–49	471,887 (54.6)	35,470 (7.5)	220,296 (51.6)	23,543 (10.7)	80,040 (54.6)	5,071 (6.3)	2,194 (91.3)	82 (3.7)	774,417 (53.8)	64,166 (8.3)
50–64	60,083 (6.9)	6,967 (11.6)	126,358 (29.6)	15,532 (12.3)	28,405 (19.4)	2,615 (9.2)	31 (1.3)	02 (6.5)	214,877 (14.9)	25,116 (11.7)
65+	5,478 (0.6)	577 (10.5)	32,036 (7.5)	5,510 (17.2)	15,550 (10.6)	2,229 (14.3)	02 (0.1)	0 (0.0)	53,066 (3.7)	8,316 (15.7)
Age of contact, y										
<16	264,267 (30.6)	19,065 (7.2)	115,376 (27.0)	14,574 (12.6)	38,419 (26.2)	3,103 (8.1)	812 (33.8)	44 (5.4)	418,874 (29.1)	36,786 (8.8)
16–24	104,631 (12.1)	8,059 (7.7)	37,680 (8.8)	2,833 (7.5)	12,263 (8.4)	623 (5.1)	145 (6.0)	03 (2.1)	154,719 (10.7)	11,518 (7.4)
25–49	287,052 (33.2)	23,550 (8.2)	120,365 (28.2)	11,148 (9.3)	43,530 (29.7)	2,337 (5.4)	948 (39.5)	30 (3.2)	451,895 (31.4)	37,065 (8.2)
50–64	170,102 (19.7)	12,419 (7.3)	106,698 (25.0)	12,493 (11.7)	34,487 (23.5)	2,528 (7.3)	361 (15.0)	6 (1.7)	311,648 (21.6)	27,446 (8.8)
≥65	38,509 (4.5)	1,664 (4.3)	46,711 (10.9)	5,931 (12.7)	17,777 (12.1)	2,011 (11.3)	136 (5.7)	2 (1.5)	103,133 (7.2)	9,608 (9.3)
Sex of index case										
M	439,408 (50.8)	33,514 (7.6)	206,392 (48.4)	25,003 (12.1)	57,613 (39.3)	4,866 (8.4)	1,260 (52.5)	42 (3.3)	704,673 (48.9)	63,425 (9.0)
F	425,153 (49.2)	31,243 (7.3)	220,438 (51.6)	21,976 (10.0)	88,863 (60.7)	5,736 (6.5)	1,142 (47.5)	43 (3.8)	735,596 (51.1)	58,998 (8.0)
Sex of contact										
M	436,910 (50.5)	28,789 (6.6)	214,572 (50.3)	20,677 (9.6)	76,160 (52.0)	4,869 (6.4)	1,173 (48.8)	40 (3.4)	728,815 (50.6)	54,375 (7.5)
F	427,651 (49.5)	35,968 (8.4)	212,258 (49.7)	26,302 (12.4)	70,316 (48.0)	5,733 (8.2)	1,229 (51.2)	45 (3.7)	711,454 (49.4)	68,048 (9.6)
Period										
Feb–Mar	238,844 (27.6)	22 (9.3)	13 (0.0)	0 (0.0)	50 (0.0)	1 (2.0)	0 (0.0)	0	238,907 (16.6)	22,101 (9.3)
Apr–May	44,026 (5.1)	2,709 (6.2)	2,215 (0.5)	88 (4.0)	2,221 (1.5)	31 (1.4)	0 (0.0)	0	48,462 (3.4)	2,828 (5.8)
Jun–Jul	323,581 (37.4)	20,182 (6.2)	172,186 (40.3)	14,896 (8.7)	48,178 (32.9)	2,808 (5.8)	167 (7.0)	1 (0.6)	544,112 (37.8)	37,887 (7.0)
Aug–Sep	258,110 (29.9)	19,766 (7.7)	252,416 (59.1)	31,995 (12.7)	96,027 (65.6)	7,762 (8.1)	2,235 (93.0)	84 (3.8)	608,788 (42.3)	59,607 (9.8)
IMD										
1 (most deprived)	261,996 (30.3)	18,754 (7.2)	79,236 (18.6)	8,521 (10.8)	27,859 (19.0)	2,060 (7.4)	311 (12.9)	12 (3.9)	369,402 (25.6)	29,347 (7.9)
2	199,652 (23.1)	14,142 (7.1)	82,520 (19.3)	8,642 (10.5)	29,795 (20.3)	2,126 (7.1)	452 (18.8)	08 (1.8)	312,419 (21.7)	24,918 (8.0)
3	157,416 (18.2)	11,684 (7.4)	85,213 (20.0)	9,305 (10.9)	29,926 (20.4)	2,130 (7.1)	515 (21.4)	24 (4.7)	273,070 (19.0)	23,143 (8.5)
4	132,905 (15.4)	10,764 (8.1)	88,346 (20.7)	9,932 (11.2)	29,664 (20.3)	2,181 (7.4)	504 (21.0)	13 (2.6)	251,419 (17.5)	22,890 (9.1)

Characteristic	Unvaccinated Contacts	Unvaccinated Secondary cases	Ch4dOx1 nCOV-19 Contacts	Ch4dOx1 nCOV-19 Secondary cases	BNT162b2 Contacts	BNT162b2 Secondary cases	mRNA-127 Contacts	mRNA-127 Secondary cases	Total Contacts	Total Secondary cases
5 (least deprived)	112,592 (13.0)	9,413 (8.4)	91,515 (21.4)	10,579 (11.6)	29,232 (20.0)	2,105 (7.2)	620 (25.8)	28 (4.5)	233,959 (16.2)	22,125 (9.5)
Household type										
Adult pair/couple	38,022 (4.4)	4,628 (12.2)	36,608 (8.6)	5,704 (15.6)	10,932 (7.5)	1,054 (9.6)	169 (7.0)	2 (1.2)	85,731 (6.0)	11,388 (13.3)
Older pair/couple	5,091 (0.6)	466 (9.2)	22,007 (5.2)	4,545 (20.7)	9,647 (6.6)	1,623 (16.8)	20 (0.8)	1 (5.0)	36,765 (2.6)	6,635 (18.0)
HH with children	459,155 (53.1)	38,217 (8.3)	180,189 (42.2)	22,360 (12.4)	59,442 (40.6)	4,598 (7.7)	1,330 (55.4)	68 (5.1)	700,116 (48.6)	65,243 (9.3)
Multigeneration HH	74,993 (8.7)	4,530 (6.0)	33,418 (7.8)	2,604 (7.8)	11,387 (7.8)	646 (5.7)	162 (6.7)	6 (3.7)	119,960 (8.3)	7,786 (6.5)
Adult only HH	287,300 (33.2)	16,916 (5.9)	154,608 (36.2)	11,766 (7.6)	55,068 (37.6)	2,681 (4.9)	721 (30.0)	8 (1.1)	497,697 (34.6)	31,371 (6.3)
Region										
North	285,797 (33.1)	23,177 (8.1)	138,426 (32.4)	16,767 (12.1)	46,974 (32.1)	3,822 (8.1)	662 (27.6)	25 (3.8)	471,859 (32.8)	43,791 (9.3)
Midlands	180,579 (20.9)	14,715 (8.1)	86,702 (20.3)	10,068 (11.6)	26,538 (18.1)	2,144 (8.1)	257 (10.7)	10 (3.9)	294,076 (20.4)	26,937 (9.2)
London	146,751 (17.0)	7,523 (5.1)	54,955 (12.9)	4,087 (7.4)	21,232 (14.5)	1,178 (5.5)	427 (17.8)	11 (2.6)	223,365 (15.5)	12,799 (5.7)
South	251,434 (29.1)	19,342 (7.7)	146,747 (34.4)	16,057 (10.9)	51,732 (35.3)	3,458 (6.7)	1,056 (44.0)	39 (3.7)	450,969 (31.3)	38,896 (8.6)
Contact vaccination										
Unvaccinated	657,785 (76.1)	52,585 (8.0)	186,797 (43.8)	17,707 (9.5)	63,610 (43.4)	3,825 (6.0)	1,264 (52.6)	51 (4.0)	909,456 (63.1)	74,168 (8.2)
Ch4dOX1 nCOV-19	141,425 (16.4)	9,088 (6.4)	167,247 (39.2)	22,548 (13.5)	42,588 (29.1)	3,455 (8.1)	584 (24.3)	21 (3.6)	351,844 (24.4)	35,112 (10.0)
BNT162b2	63,776 (7.4)	3,046 (4.8)	70,788 (16.6)	6,637 (9.4)	39,503 (27.0)	3,296 (8.3)	318 (13.2)	06 (1.9)	174,385 (12.1)	12,985 (7.4)
mRNA-127	1,575 (0.2)	38 (2.4)	1,998 (0.5)	87 (4.4)	775 (0.5)	26 (3.4)	236 (9.8)	7 (3.0)	4,584 (0.3)	158 (3.4)
Total	864,561 (100)	64,757 (7.5)	426,830 (100)	46,979 (11.0)	146,476 (100)	10,602 (7.2)	2,402 (100)	85 (3.5)	1,440,269 (100)	122,423 (8.5)

*HH, household; IMD, Index of Multiple Deprivation.
